# Antimicrobial Resistance in *Neisseria gonorrhoeae* Isolates from Men with Urethral or Urogenital Infection: A Systematic Review and Meta-Analysis

**DOI:** 10.3390/medicina62081457

**Published:** 2026-07-28

**Authors:** Daurenbek Sengirbayev, Marat Batyrbekov, Zhakhanger Tursunov, Ulugbek Medeubekov, Tilek Kassymov, Mustafa Rysuly, Indira Karibayeva, Zhansaya Sultanbayeva

**Affiliations:** 1 Urology Department, Asfendiyarov Kazakh National Medical University, 050012 Almaty, Kazakhstan; dausengir@mail.ru (D.S.); marat.batyrbekov@mail.ru (M.B.); 2 Science Department, Kazakhstan’s Medical University “KSPH”, 050060 Almaty, Kazakhstan; 3 Science Department, Kazakh Scientific Center of Dermatology and Infectious Diseases, 050008 Almaty, Kazakhstan; medeubek@mail.ru (U.M.); resulymustafa@gmail.com (M.R.); 4 Scientific Center of Urology named after Academician B.U. Dzharbusynov, 050060 Almaty, Kazakhstan; kassymov97@gmail.com; 5 Jiann-Ping Hsu College of Public Health, Georgia Southern University, Statesboro, GA 30460, USA; ik01379@georgiasouthern.edu; 6 Science Department, Kazakh Eye Research Institute, 050012 Almaty, Kazakhstan; zh.sultanbaeva@eyeinst.kz

**Keywords:** *Neisseria gonorrhoeae*, antimicrobial resistance, meta-analysis, urogenital infection, male population, antibiotic susceptibility

## Abstract

*Background and Objectives*: Antimicrobial resistance in *Neisseria gonorrheae* threatens the effectiveness of empirical gonorrhoea treatment. Although global surveillance evidence is increasingly available, resistance data are not consistently reported separately for men with urethral or urogenital infection. This review evaluated antibiotic-specific resistance in *N. gonorrhoeae* isolates from this clinically defined population and examined the geographic and reporting limitations of the available evidence. *Materials and Methods*: PubMed, Scopus, ScienceDirect, Google Scholar, and relevant WHO GASP/EGASP resources were searched from inception to [updated search date]. Eligible observational or surveillance studies reported categorical resistance classifications or extractable antibiotic-specific resistant counts and denominators for male urethral or urogenital isolates. Antibiotic-specific proportions were synthesized using random-effects meta-analysis where pooling was considered appropriate. Sparse-event outcomes were additionally evaluated using study-specific estimates and aggregate resistant/tested counts. Certainty of evidence was assessed using the GRADE framework adapted for prevalence outcomes. *Results*: Six studies met the eligibility criteria, including the 2025 multinational EGASP study. Cefixime resistance was reported in 748 of 13,154 isolates, corresponding to an aggregate proportion of 5.69%; however, the random-effects estimate was unstable because three studies reported no resistant isolates and events were concentrated in two large datasets. Ceftriaxone resistance varied from 0% to 5.17%, with a pooled estimate of 0.32% (95% CI 0.05–2.15; I^2^ = 97.8%). Resistance was high but heterogeneous for ciprofloxacin (71.84%, 95% CI 7.53–98.77; I^2^ = 97.3%) and penicillin (62.47%, 95% CI 36.87–82.58; I^2^ = 98.1%). Spectinomycin resistance was 2.57% (95% CI 1.58–4.16; I^2^ = 0%), while azithromycin resistance was 6.38% (95% CI 4.38–9.21; I^2^ = 94.9%). Certainty of evidence was very low for all outcomes. *Conclusions*: Evidence specifically reporting resistance in male urethral or urogenital *N. gonorrhoeae* isolates remains limited and geographically uneven. Contemporary EGASP data demonstrate that ceftriaxone resistance is not uniformly low across surveillance settings. The pooled estimates should be interpreted as exploratory summaries rather than current global prevalence values or an independent basis for treatment selection. Standardized reporting of sex, anatomical site, breakpoints, MIC distributions, and resistant/tested counts is needed to improve future surveillance synthesis.

## 1. Introduction

Gonorrhea remains one of the most prevalent bacterial sexually transmitted infections (STIs) worldwide and continues to represent a significant global public health concern. Despite the availability of effective antimicrobial therapy, *Neisseria gonorrhoeae* infection persists as a challenging and controversial disease with respect to optimal treatment strategies [1]. According to the World Health Organization (WHO), an estimated 82.4 million new gonorrhea infections occurred globally among adults in 2020 alone, highlighting the substantial and ongoing burden of disease across both high-income and low- and middle-income countries [2]. The clinical and epidemiological importance of gonorrhea is further amplified by its association with severe reproductive and neonatal complications, including pelvic inflammatory disease, infertility, ectopic pregnancy, and facilitation of human immunodeficiency virus (HIV) transmission [3,4]. Notably, HIV transmission among men has increased in recent years, particularly in the Eastern Europe and Central Asia (EECA) region, where HIV prevalence continues to rise while declining in most other parts of the world [5].

A major challenge in contemporary gonorrhea control is the rapid emergence and global spread of antimicrobial resistance (AMR) [3,6]. *Neisseria gonorrhoeae* has increasingly developed resistance to nearly all antibiotics previously used for treatment, including sulfonamides, penicillins, tetracyclines, fluoroquinolones, macrolides, and, more recently, extended-spectrum cephalosporins. This evolving resistance has complicated empirical therapy and necessitated repeated revisions of international treatment guidelines [7]. Recent surveillance data from nine sentinel countries participating in the WHO Enhanced Gonococcal Antimicrobial Surveillance Programme (EGASP) in 2023 underscore the severity of the problem. Among 2491 gonococcal isolates, resistance was observed in 3.8% to ceftriaxone, 8.9% to cefixime, 3.6% to azithromycin, and 95.3% to ciprofloxacin, with particularly high ceftriaxone resistance reported in Cambodia (15.3%) and Viet Nam (20.4%) [8]. These findings highlight the growing threat to first-line therapies and reinforce the urgent need for strengthened surveillance systems and the development of novel antimicrobial agents [9,10].

Previous systematic reviews have provided valuable broad assessments of gonococcal antimicrobial resistance but have commonly combined women and men, multiple anatomical sites, symptomatic and asymptomatic infections, and heterogeneous surveillance populations [11]. Although such syntheses characterize broad resistance patterns, they do not establish whether sufficiently stratified evidence is available for men with urethral or urogenital infection, a population in whom culture-based antimicrobial susceptibility testing is frequently undertaken.

Focusing specifically on male urogenital isolates addresses an important gap in the literature. In men, gonorrhea more commonly presents as symptomatic urethritis, which is associated with earlier healthcare-seeking behaviour, higher likelihood of microbiological confirmation, and greater feasibility of culture-based antimicrobial susceptibility testing [12]. In contrast, women more often have asymptomatic or nonspecific infections, which may lead to delayed diagnosis, underrepresentation in culture-based surveillance, and differences in observed resistance patterns [13]. Similarly, combining urogenital, rectal, and pharyngeal specimens may obscure clinically relevant differences, as anatomical site influences bacterial load, diagnostic yield, treatment response, and opportunities for resistance selection.

The present review therefore aimed to: (1) identify antibiotic-specific resistance data for *N. gonorrhoeae* isolates obtained from men with urethral or urogenital infection; (2) conduct exploratory antibiotic-specific meta-analyses when the available evidence was sufficiently comparable; and (3) characterize the geographic, temporal, and reporting gaps that limit interpretation of sex- and specimen-specific surveillance data. The review was not designed to estimate contemporary global resistance prevalence.

## 2. Materials and Methods

### 2.1. Search Strategy

The protocol was prospectively registered in PROSPERO (CRD420261284374) [14]. The original systematic search was conducted in October 2025 and was updated in July 2026 using PubMed, Scopus, ScienceDirect, and Google Scholar. A targeted supplementary search of WHO EGASP resources was also undertaken, and the reference lists of eligible studies and relevant surveillance reports were screened. The complete database-specific search strategies are provided in Supplementary Table S1.

### 2.2. Eligibility Criteria

Studies were eligible if they met prespecified criteria based on the Population, Intervention, Comparator, Outcome, and Study Design (PICOS) framework (Supplemental Table S2). The population comprised men with laboratory-confirmed urethral or urogenital *N. gonorrhoeae* infection from whom urethral or other clearly defined male urogenital isolates were obtained. No intervention or comparator was applicable because this review synthesized AMR. Studies were included if they: (1) reported antimicrobial susceptibility and/or resistance outcomes for *N. gonorrhoeae* isolates from the target population; (2) used an appropriate laboratory method for antimicrobial susceptibility testing (AST); and (3) provided sufficient data to extract or calculate resistance/susceptibility estimates for at least one antibiotic of interest. Eligible designs included cross-sectional studies, prospective or retrospective observational studies, and surveillance studies/reports with extractable primary data; cohort and case–control studies were also included when relevant AMR outcomes were reported.

Studies were excluded if they were reviews, editorials, commentaries, case reports, or conference abstracts without full extractable data; did not report male-specific results; did not include urogenital or urethral specimens; lacked extractable antimicrobial susceptibility or resistance outcomes; or used non-standard, unclear, or insufficiently described susceptibility methods that precluded interpretation. Studies were also excluded when resistance data for male urogenital or urethral isolates could not be separated from female participants, mixed-sex populations, or non-urogenital anatomical sites, including rectal or pharyngeal specimens. Reports presenting only MIC ranges, MIC_50_, MIC_90_, or graphical MIC distributions were excluded from quantitative synthesis when antibiotic-specific categorical resistance classifications or resistant counts and denominators could not be extracted. Studies using MIC-based AST methods were eligible when recognized interpretive breakpoints were applied, and categorical resistant counts were reported or could be calculated.

### 2.3. Study Selection and Data Collection

Study selection and data collection were conducted in accordance with PRISMA recommendations (Supplemental Table S3) [15]. After database searching, all retrieved records were imported into the Mendeley reference management workflow, and duplicates were removed. Three reviewers (Z.T., I.K., Z.S.) independently screened unique records by title and abstract, followed by full-text review to confirm eligibility against the predefined inclusion criteria. Any discrepancies in study selection or data extraction were resolved through discussion and, when necessary, adjudication by a third reviewer, with consensus reached for all included studies.

Data were extracted using a standardized form. Extracted variables included: first author, year of publication, country/region, study period, study design, setting and sampling frame, sample size (overall and number tested per antibiotic), specimen type (urogenital swab), diagnostic method for gonorrhea, AST methodology (e.g., agar dilution, Etest, disc diffusion), antibiotics tested, and counts/proportions classified as susceptible/intermediate/resistant (or non-susceptible/decreased susceptibility). When multiple reports described overlapping populations, we retained the most recent or most informative dataset.

For quantitative synthesis, studies were required to provide categorical antimicrobial susceptibility results, including susceptible, intermediate, and resistant classifications, or sufficient data to extract antibiotic-specific resistant counts and denominators. During the updated review, contemporary GASP and EGASP reports were assessed using the same prespecified eligibility criteria. Reports were evaluated for overlap according to countries, sentinel sites, surveillance periods, and participant populations. Only the most comprehensive nonoverlapping dataset was included in the quantitative synthesis. At title and abstract screening, records were excluded when they clearly did not concern phenotypic gonococcal antimicrobial resistance, did not include the prespecified male urethral or urogenital population, focused exclusively on treatment, diagnostic accuracy, genomic mechanisms, or laboratory methodology without extractable clinical resistance data, or represented an ineligible publication type. Specific reasons were recorded for all reports excluded after full-text assessment.

### 2.4. Outcome Measures

The primary outcomes were defined as antibiotic-specific resistance proportions for each antimicrobial agent with sufficient extractable data. Resistance was analyzed separately for each antibiotic, given the differences in therapeutic indications, mechanisms of resistance, antimicrobial susceptibility testing methods, and interpretive breakpoints across agents. Accordingly, an overall pooled estimate combining resistance across different antibiotics was not calculated, as such a measure would have limited clinical and methodological interpretability. Where data were sufficiently comparable, pooled resistance estimates were evaluated separately for cefixime, ceftriaxone, ciprofloxacin, penicillin, spectinomycin, and azithromycin. Antimicrobial agents with insufficient or non-comparable data were summarized descriptively, particularly when studies differed in reporting format, susceptibility testing methodology, breakpoint criteria, or availability of antibiotic-specific denominators and resistant counts.

MIC distributions were not pooled directly with categorical resistance proportions. However, studies using MIC-based testing were eligible when established interpretive breakpoints were applied, and antibiotic-specific resistant counts and denominators were reported or extractable.

### 2.5. Statistical Analysis

Antibiotic-specific resistance was calculated as the number of resistant isolates divided by the number tested for the corresponding antimicrobial agent. Study-specific proportions were presented with 95% confidence intervals. Where multiple nonoverlapping subgroups were reported within the same study, subgroup counts were combined before the primary study-level meta-analysis to avoid treating correlated strata as independent studies.

Antibiotic-specific proportions were synthesized using a random-effects model after logit transformation of proportions; therefore, pooled estimates were calculated on the logit scale, with between-study variance estimated using maximum likelihood. Zero-event datasets were retained in the analysis. For sparse-event outcomes, the model-based estimate was interpreted together with study-specific estimates and the aggregate number resistant divided by the aggregate number tested. The aggregate proportion was reported descriptively and was not considered equivalent to the random-effects estimate.

No pooled estimate was considered substantively interpretable when model instability, sparse events, extreme heterogeneity, or concentration of events in a small number of datasets resulted in a summary estimate that did not adequately represent the observed study-specific evidence. Such outcomes were emphasized descriptively and, where appropriate, the model-based estimate was retained only as a sensitivity analysis.

Heterogeneity was assessed using Cochran’s Q, τ^2^, and I^2^. Because fewer than ten independent studies contributed to each outcome, funnel plots and regression-based assessments of small-study effects were not performed. All statistical analyses were performed using R software, version 4.5.1 (R Foundation for Statistical Computing, Vienna, Austria), within the RStudio IDE (Posit Software, PBC, Boston, MA, USA) [16].

### 2.6. Quality Assessment and Certainty of Evidence

Methodological quality and risk of bias were assessed independently by two reviewers using the Joanna Briggs Institute Critical Appraisal Checklist for Studies Reporting Prevalence Data (Supplemental Table S4). This appraisal focused on sampling representativeness, validity and reliability of outcome measurement (including AST methodology and interpretive criteria), and completeness and transparency of reporting. Disagreements were resolved through discussion and, if needed, third-reviewer adjudication.

The overall certainty of evidence for each antibiotic-specific pooled estimate was evaluated using the GRADE approach adapted for prevalence outcomes. Certainty was assessed across five domains: risk of bias, inconsistency (based on I^2^ and overlap of study estimates), indirectness (alignment with PICOS and specimen type), imprecision (precision of pooled 95% CI and sample size), and publication bias (funnel plots and Egger’s test where applicable). Because all included evidence originated from observational or surveillance studies, certainty initially started at low and was subsequently downgraded for serious or very serious concerns regarding risk of bias, inconsistency, indirectness, imprecision, or publication bias. Publication bias was not formally assessed when fewer than ten independent studies contributed to an outcome.

## 3. Results

### 3.1. Study Selection and Characteristics of the Included Studies

A total of 483 records were identified through database and register searching, including PubMed (*n* = 161), Scopus (*n* = 54), ScienceDirect (*n* = 252), Google Scholar (*n* = 13), and WHO EGASP sources (*n* = 3). After removal of 58 duplicate records, 425 records were screened by title and abstract. Of these, 363 records were excluded because they were not directly relevant to the study objective, primarily due to a focus on treatment approaches, clinical guidelines, diagnostic methods, genomic-only investigations, or the absence of extractable phenotypic antimicrobial susceptibility testing data for male urogenital *N. gonorrhoeae* isolates.

A total of 62 reports were sought for retrieval; the full text could not be obtained for one report. Consequently, 61 full-text reports were assessed for eligibility. Following full-text review, 55 reports were excluded for the following reasons: the gonorrhea sample size was reported only as a total without sex stratification (*n* = 37), no extractable resistance information was provided (*n* = 4), only MIC-based outcomes were reported without categorical S/I/R classification or extractable resistant counts (*n* = 8), earlier EGASP reports were excluded to avoid duplication with the most recent included EGASP dataset (*n* = 2), no numerical data were available (*n* = 1) [17], AMR data were reported jointly for multiple infections without separate gonorrhoea-specific results (*n* = 1) [18], urethral and oral swab results were combined without separable data (*n* = 1), and urethral and rectal swab results were combined without separable data (*n* = 1) [19].

**Figure 1:**
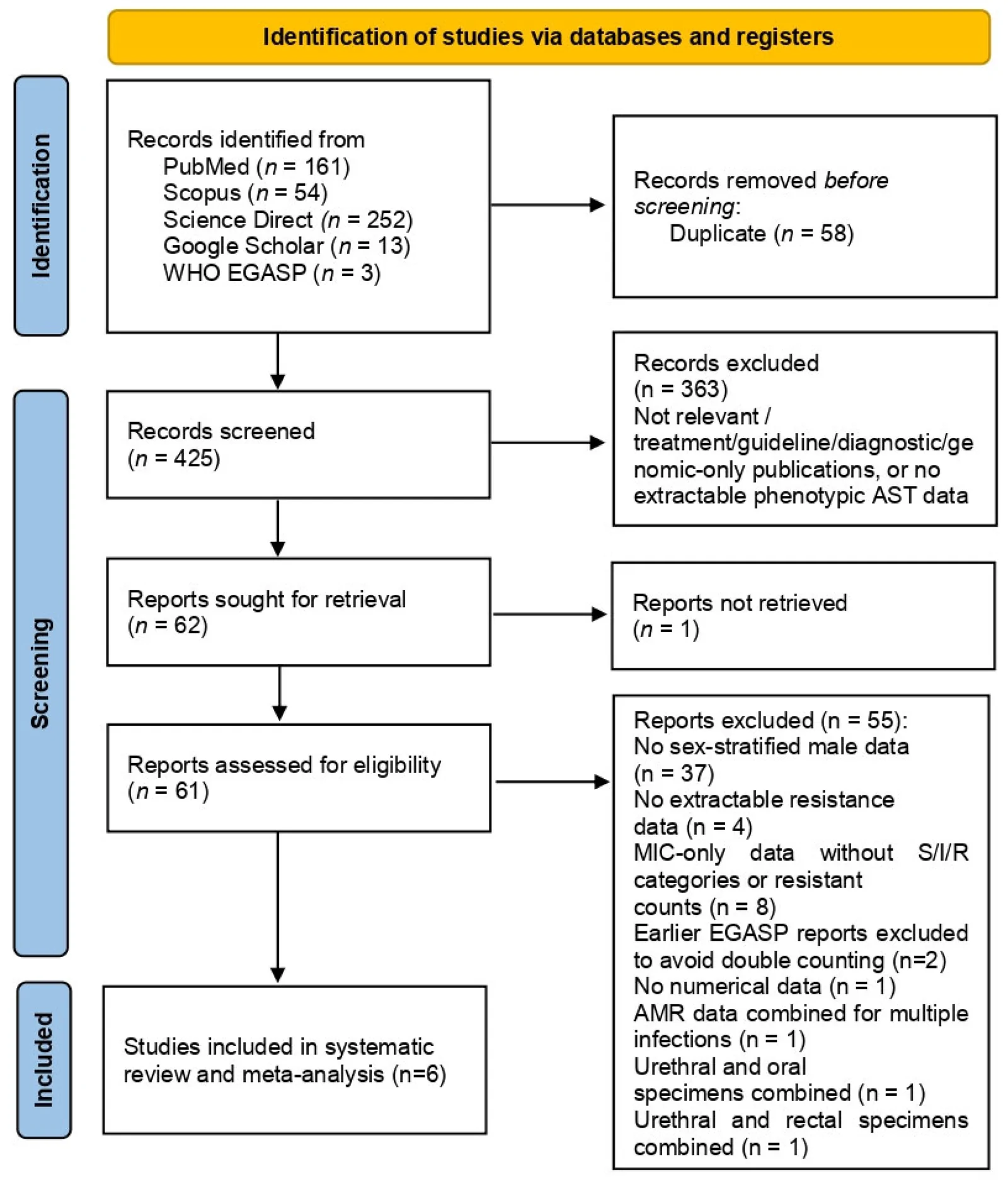
PRISMA flow diagram of study selection.

In total, six studies or surveillance datasets met all eligibility criteria and were included in the systematic review and quantitative meta-analysis. The PRISMA flow diagram illustrating the study selection process is presented in Figure 1.

The six included studies were published between 2003 and 2025 and represented observational or surveillance data from the WHO African, Eastern Mediterranean, European, South-East Asian, and Western Pacific regions. Geographic coverage remained uneven, and no eligible sex- and specimen-stratified study from the Region of the Americas was identified. AST was performed using disc diffusion or MIC-based methods, including Etest, with study-specific interpretive criteria. The numbers tested differed substantially by antibiotic and study; resistance estimates were therefore analyzed separately for each antimicrobial agent. Detailed characteristics of the included studies are presented in Table 1.

### 3.2. Meta-Analysis of Antibiotic Resistance

**Figure 2:**
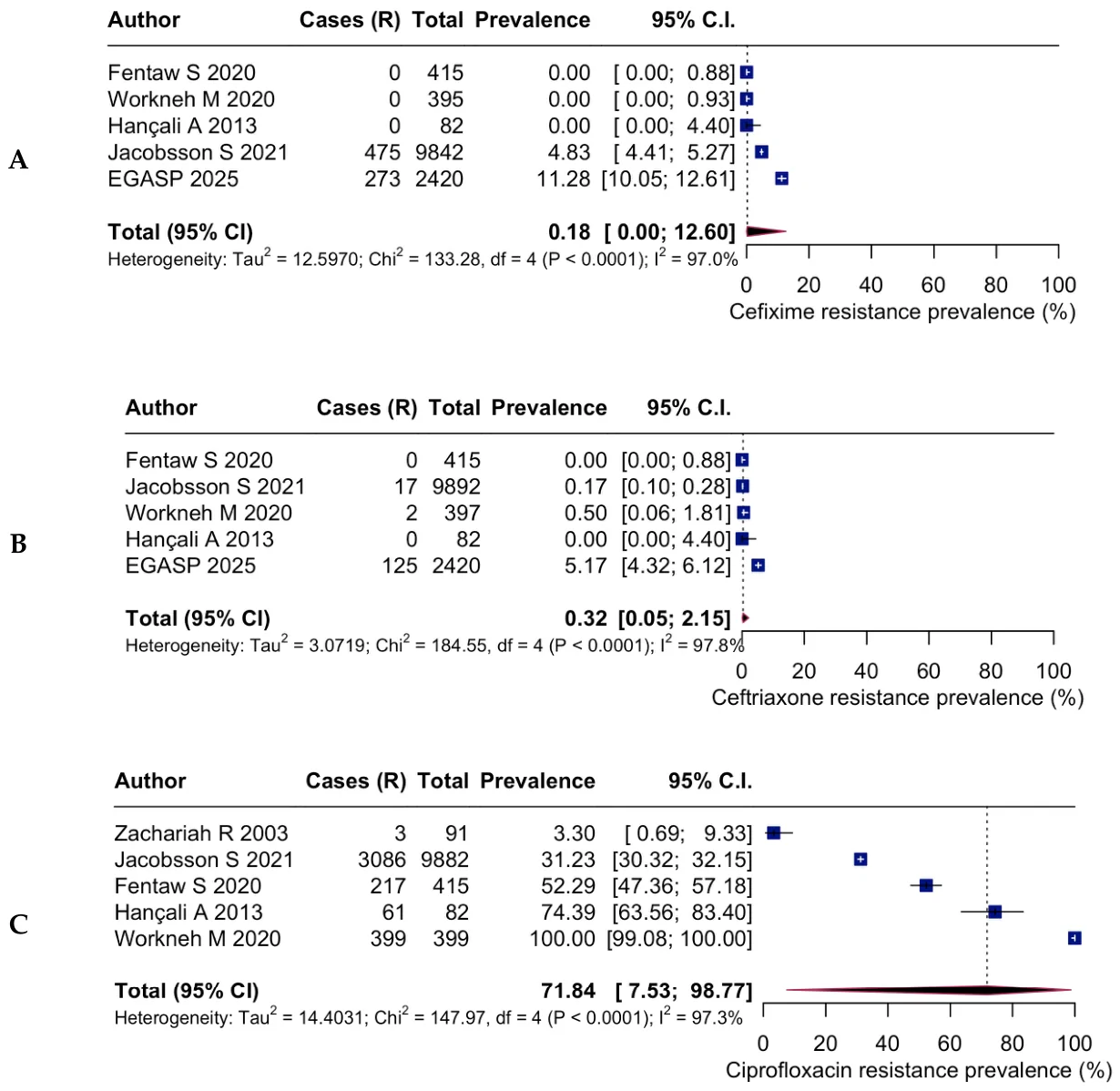

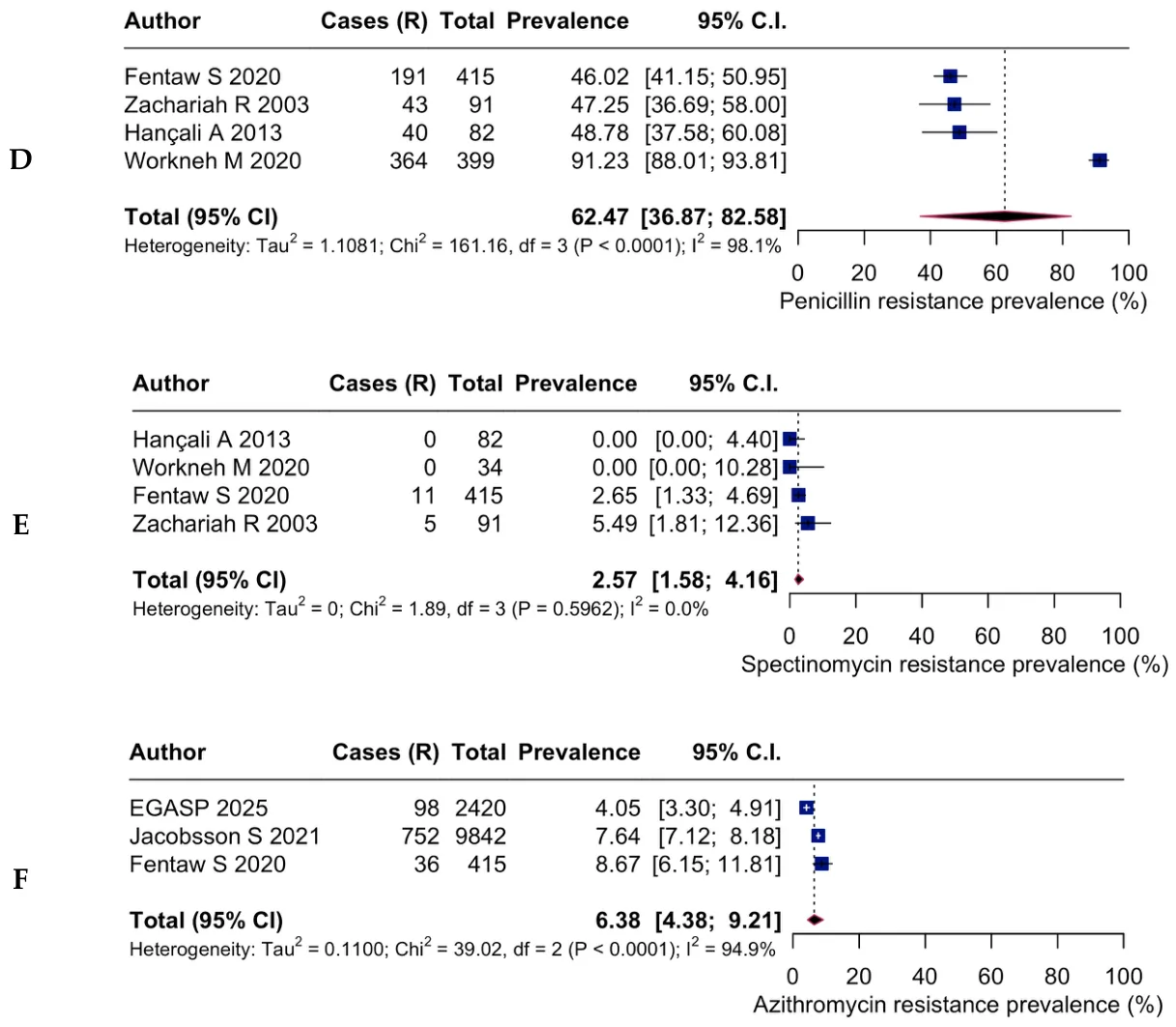
Random-effects meta-analysis of antimicrobial resistance in male urethral or urogenital *Neisseria gonorrhoeae* isolates: (**A**) cefixime; (**B**) ceftriaxone; (**C**) ciprofloxacin; (**D**) penicillin; (**E**) spectinomycin; and (**F**) azithromycin. Studies included in the meta-analysis: Zachariah, 2003 [20], Hancali, 2013 [21], Fentaw, 2020 [22], Workneh, 2020 [23], Jacobson, 2021 [24], EGASP, 2025 [8]. Abbreviations: CI—confidence interval.

The antibiotic-specific meta-analyses of resistance in *Neisseria gonorrhoeae* isolates obtained from men with urogenital or urethral infection are presented in Figure 2. Resistance was analyzed separately for each antimicrobial agent because the contributing studies differed in geographic setting, surveillance period, antimicrobial susceptibility testing methods, interpretive breakpoints, and the number of isolates tested.

Cefixime resistance was evaluated across five datasets comprising 13,154 isolates, of which 748 were resistant, corresponding to an aggregate proportion of 5.69%. Three studies reported no cefixime-resistant isolates: Fentaw et al. [22], Workneh et al. [23], and Hançali et al. [21]. Jacobsson et al. reported resistance in 475 of 9842 isolates (4.83%) [24], whereas the contemporary EGASP [25] dataset reported resistance in 273 of 2420 isolates (11.28%) [8]. The random-effects model produced a pooled resistance proportion of 0.18% (95% CI: 0.00–12.60). Between-study heterogeneity was considerable (I^2^ = 97.0%; *p* < 0.0001). The marked discrepancy between the aggregate proportion and the model-based estimate, together with the coexistence of several zero-event studies and two large non-zero datasets, indicates substantial statistical instability. The pooled cefixime estimate should therefore not be interpreted as a single representative resistance prevalence.

Ceftriaxone resistance was assessed across five datasets including 13,206 isolates, with 144 resistant isolates overall, corresponding to an aggregate proportion of 1.09%. No resistant isolates were reported by Fentaw et al. [22] or Hançali et al. [21]. Resistance was 0.17% in Jacobsson et al. [24] and 0.50% in Workneh et al. [23]. In contrast, the EGASP dataset reported 125 resistant isolates among 2420 tested isolates, corresponding to 5.17% resistance [8]. The pooled resistance proportion was 0.32% (95% CI: 0.05–2.15), with considerable heterogeneity (I^2^ = 97.8%; *p* < 0.0001). These findings indicate that ceftriaxone resistance was not consistently low across settings and that the overall estimate was strongly influenced by geographic and temporal differences between earlier studies and contemporary EGASP surveillance.

Ciprofloxacin resistance was evaluated across five studies comprising 10,869 isolates, of which 3766 were resistant. The pooled resistance proportion was 71.84% (95% CI: 7.53–98.77), with considerable between-study heterogeneity (I^2^ = 97.3%; *p* < 0.0001). Study-specific estimates ranged from 3.30% in Zachariah et al. [20] to 100% in Workneh et al. [23]. Intermediate estimates included 31.23% in Jacobsson et al. [24], 52.29% in Fentaw et al. [22], and 74.39% in Hançali et al. [21]. The wide confidence interval and marked variability across studies indicate that the pooled estimate represents an average across highly heterogeneous surveillance settings rather than a universally applicable resistance proportion.

Penicillin resistance was assessed across four studies including 987 isolates, of which 638 were resistant. The pooled resistance proportion was 62.47% (95% CI: 36.87–82.58), with considerable heterogeneity (I^2^ = 98.1%; *p* < 0.0001). Study-specific resistance ranged from 46.02% in Fentaw et al. [22] to 91.23% in Workneh et al. [23]. Zachariah et al. [20] and Hançali et al. [21] reported resistance proportions of 47.25% and 48.78%, respectively. These findings demonstrate consistently substantial penicillin resistance, although its magnitude differed markedly between settings.

Spectinomycin resistance was evaluated across four studies comprising 622 isolates, with 16 resistant isolates. The pooled resistance proportion was 2.57% (95% CI: 1.58–4.16). No statistical heterogeneity was detected (I^2^ = 0.0%; *p* = 0.5962). Hançali et al. [21] and Workneh et al. [23] reported no resistant isolates, whereas resistance was 2.65% in Fentaw et al. [22] and 5.49% in Zachariah et al. [20]. Although the estimates were statistically consistent, the small number of studies and limited number of tested isolates restrict the precision and generalizability of this finding.

Azithromycin resistance was assessed using three independent study-level datasets after combining non-overlapping subgroups reported within the same study. Overall, 886 resistant isolates were identified among 12,677 tested isolates, corresponding to an aggregate resistance proportion of 6.99%. Resistance was 4.05% in the EGASP dataset [8], 7.64% in Jacobsson et al. [24], and 8.67% in Fentaw et al. [22]. The pooled resistance proportion was 6.38% (95% CI: 4.38–9.21). Despite the relatively narrow range of point estimates, statistical heterogeneity was considerable (I^2^ = 94.9%; *p* < 0.0001), indicating that differences in study size, precision, surveillance settings, study periods, or susceptibility-testing criteria contributed to variability between estimates.

Accordingly, all pooled estimates were interpreted at the individual-antibiotic level. Particular caution is required for cefixime and ceftriaxone because the updated EGASP data materially changed the distribution of resistance events and revealed substantial heterogeneity that was not apparent in the earlier evidence base.

Leave-one-out sensitivity analysis showed that the stability of pooled estimates differed across antibiotics (Table 2). For azithromycin, omission of EGASP [8] reduced heterogeneity to 0%, indicating that this dataset was the main contributor to between-study variability. A similar pattern was observed for ceftriaxone, where exclusion of EGASP [8] also reduced heterogeneity to 0%, suggesting a strong influence of the recent surveillance dataset on the pooled estimate.

For cefixime, the results were unstable: omission of Jacobsson [24] or EGASP [8] markedly changed the pooled estimate and reduced heterogeneity to 0%, indicating that resistant events were concentrated mainly in these large non-zero datasets. Ciprofloxacin remained highly heterogeneous regardless of which study was omitted, suggesting that variability was not driven by a single study but reflected genuine geographic and temporal differences.

For penicillin, omission of Workneh [23] reduced heterogeneity to 0%, identifying it as an influential study in this pool. In contrast, spectinomycin estimates remained stable across all leave-one-out iterations, with heterogeneity consistently at 0%, supporting the robustness of the low resistance estimate, although the number of studies and resistant events was limited.

Overall, the sensitivity analysis supports the main findings but confirms that cefixime and ceftriaxone estimates should be interpreted cautiously because they are strongly influenced by recent large surveillance datasets, especially EGASP [8].

The certainty of evidence for antibiotic-specific resistance estimates was evaluated using the GRADE approach adapted for prevalence outcomes. Because all included evidence was derived from observational or surveillance studies, each outcome initially received a low-certainty rating. After consideration of inconsistency and imprecision, the certainty of evidence was rated as very low for all six antibiotics. Detailed judgments are presented in Table 3.

For ceftriaxone resistance, certainty was downgraded for serious inconsistency and serious imprecision. The updated analysis showed considerable heterogeneity (I^2^ = 97.8%), with study-specific resistance ranging from 0% to 5.17% and most resistant isolates contributed by the contemporary EGASP dataset. Cefixime resistance was downgraded for serious inconsistency and very serious imprecision because three studies reported no resistant isolates, resistance events were concentrated in two large datasets, and the model-based pooled estimate remained unstable, with a wide confidence interval.

Certainty for ciprofloxacin resistance was rated as very low because of serious inconsistency and very serious imprecision. Study-specific resistance estimates ranged from 3.30% to 100%, heterogeneity was considerable (I^2^ = 97.3%), and the pooled confidence interval was extremely wide. Penicillin resistance was downgraded for serious inconsistency because heterogeneity was substantial (I^2^ = 98.1%) and study-specific estimates ranged from 46.02% to 91.23%. No additional downgrade for imprecision was applied because the entire confidence interval indicated a substantial resistance burden.

Spectinomycin resistance was downgraded for serious imprecision. Although no statistical heterogeneity was detected (I^2^ = 0%), the estimate was based on only four studies, 622 tested isolates, and 16 resistance events. Azithromycin resistance was downgraded for serious inconsistency because heterogeneity was considerable (I^2^ = 94.9%), despite a comparatively narrow range of study-specific estimates from 4.05% to 8.67%. No downgrade for imprecision was applied to azithromycin because the pooled estimate was supported by a large total sample and had a relatively precise confidence interval.

Risk of bias and indirectness were not considered serious for any outcome because the included studies, including EGASP, used appropriate surveillance or observational methods and were aligned with the prespecified population, specimen type, and resistance outcomes. Publication bias could not be formally assessed because fewer than ten independent studies contributed to each antibiotic-specific analysis. Overall, the very low certainty ratings indicate that the pooled estimates should be interpreted as exploratory summaries of the available evidence rather than precise or globally representative resistance prevalence estimates.

## 4. Discussion

### 4.1. Principal Findings and Interpretation

This systematic review identified six studies reporting antibiotic-specific resistance in *Neisseria gonorrhoeae* isolates obtained from men with urethral or urogenital infection. Incorporation of the 2025 multinational Enhanced Gonococcal Antimicrobial Surveillance Programme study extended the evidence base through 2025, added contemporary surveillance data from South-East Asian and Western Pacific settings, and materially changed the interpretation of extended-spectrum cephalosporin resistance [8]. Nevertheless, the evidence remained unevenly distributed across regions and surveillance periods. The estimates should therefore be interpreted as exploratory summaries of the eligible sex- and specimen-specific evidence rather than as contemporary global resistance prevalence estimates.

Resistance to ciprofloxacin and penicillin was generally high, but the magnitude varied substantially across settings. Ciprofloxacin resistance ranged from 3.30% to 100%, while penicillin resistance ranged from 46.02% to 91.23%. These results are consistent with the broader global literature showing persistent resistance to antimicrobial agents that were previously used widely for *gonorrhea* treatment [1,11]. However, the very high heterogeneity in the present analyses indicates that the pooled estimates do not describe a single underlying resistance level. Instead, they reflect marked geographic and temporal variation associated with antimicrobial exposure, local transmission networks, circulating gonococcal lineages, surveillance design, and laboratory methodology. Recent genomic surveillance in Europe and Brazil has similarly demonstrated that resistance patterns and dominant lineages can change substantially across relatively short periods and differ between surveillance settings [25,26].

The updated ceftriaxone analysis is particularly important because it changes the conclusion of the previous manuscript. Earlier eligible studies reported resistance proportions between 0% and 0.50%, whereas resistance in the included EGASP dataset was 5.17% [8,21,22,23,24]. The resulting pooled estimate of 0.32% should not be interpreted independently of the study-specific results because heterogeneity was considerable and most resistant isolates were contributed by the most recent surveillance dataset. Contemporary evidence outside the included meta-analysis also demonstrates the emergence and international dissemination of ceftriaxone-resistant FC428-related lineages in the Asia-Pacific region [27]. In Hanoi, 27% of isolates collected during 2023–2024 were reported as ceftriaxone-resistant, while surveillance in England identified ceftriaxone-resistant infections associated predominantly with travel from the Asia-Pacific region [28,29]. These observations indicate that a low pooled average across geographically and temporally diverse studies can obscure clinically important resistance concentrations in particular countries and transmission networks.

The cefixime analysis further illustrates the limitations of pooling sparse and highly unbalanced resistance data. Three studies reported no cefixime-resistant isolates, whereas resistance was 4.83% in Jacobsson et al. and 11.28% in EGASP [8,21,22,23,24]. The aggregate proportion was 5.69%, but the random-effects model produced an estimate of 0.18% with a 95% confidence interval extending from approximately 0% to 12.60%. This discrepancy reflects the combination of multiple zero-event datasets, large differences in study size, substantial heterogeneity, and concentration of resistant events in two large studies. The model-based estimate should therefore not be interpreted as a representative cefixime prevalence. For this outcome, the study-specific estimates and resistant/tested counts are more informative than the pooled mean.

Azithromycin resistance was estimated at 6.38%, with study-specific estimates ranging from 4.05% to 8.67%. Although this absolute range was narrower than that observed for ciprofloxacin, penicillin, cefixime, or ceftriaxone, statistical heterogeneity remained considerable. This apparent contrast is partly attributable to the high precision of the large surveillance datasets: when within-study confidence intervals are narrow, even moderate absolute differences can produce a high I^2^ value. Accordingly, the absolute resistance range, surveillance period, breakpoint criteria, and setting should be considered alongside I^2^ when interpreting azithromycin resistance. The result indicates that azithromycin resistance was present across all three contributing datasets, but it does not establish a universally applicable prevalence.

Spectinomycin resistance remained uncommon in the included studies, with a pooled estimate of 2.57% and no detected statistical heterogeneity. This finding is broadly compatible with the recent global meta-analysis by Hooshiar et al., which reported comparatively low resistance to spectinomycin relative to several historically used antibiotics [11]. However, the present estimate was based on only 622 isolates and 16 resistance events. The absence of detected heterogeneity across four small studies should not be interpreted as evidence of universal susceptibility, and the very low certainty rating precludes strong clinical conclusions.

Taken together, the antibiotic-specific findings demonstrate why resistance prevalence should not be summarized across different antimicrobial agents and why pooled means should not be interpreted without their geographic, temporal, and laboratory context. The included studies used different susceptibility-testing methods, breakpoint standards, surveillance periods, and sampling approaches. Genomic surveillance has additionally shown that local changes in the prevalence of particular lineages can alter population-level susceptibility patterns independently of broader international trends [25,26]. These factors likely explain much of the observed heterogeneity, but the small number of eligible studies prevented formal assessment through meta-regression or sufficiently powered subgroup analyses.

The principal scientific contribution of this review is therefore not the generation of a definitive global resistance estimate. Rather, it demonstrates the scarcity of published surveillance evidence reporting resistant counts simultaneously stratified by sex and anatomical specimen site. Although large national and international programmes generate substantial gonococcal AMR data, many reports combine sexes, clinical presentations, or anatomical sites in ways that prevent extraction of results specifically for male urethral infection. This restriction explains both the small evidence base and the absence of an eligible dataset from the Region of the Americas, despite the existence of contemporary gonococcal surveillance in countries such as Brazil [26]. The review consequently identifies a reporting gap that limits clinically defined evidence synthesis and comparison between surveillance systems.

From a clinical and public-health perspective, the present results should not be used independently to select empirical treatment. Ceftriaxone remains central to contemporary gonorrhea management, but emerging resistance and documented treatment failures require continued susceptibility surveillance, culture and AST in suspected treatment failure, test-of-cure where recommended, and prompt public-health investigation of resistant infections [30,31]. The updated ceftriaxone findings are therefore not an argument to abandon current recommended therapy; rather, they show that historical or globally averaged estimates cannot substitute for current national and local surveillance.

Future surveillance reports should present antibiotic-specific resistant and tested counts together with MIC distributions, interpretive breakpoints and versions, sex, anatomical specimen site, clinical presentation, country, sentinel site, and surveillance period. Reporting both categorical resistance and MIC distributions is important because shifts in MICs can precede increases in breakpoint-defined resistance. International use of quality-assured laboratory methods and reference strains is also necessary to improve comparability across time and settings [32]. Standardized reporting would enable more informative subgroup analyses, reduce avoidable exclusions from systematic reviews, and support earlier detection of emerging resistance.

### 4.2. Limitations

Several limitations affect the interpretation of this review. The evidence base remained small and geographically uneven despite inclusion of the contemporary EGASP study. Although South-East Asian and Western Pacific settings were newly represented, no eligible study from the Region of the Americas provided resistance data separately for the prespecified male urethral or urogenital population. The included studies should therefore not be considered a probability sample of global gonococcal resistance.

The surveillance periods extended from the early 2000s to 2023, during which resistance patterns, testing methods, interpretive breakpoints, antimicrobial use, and circulating gonococcal lineages changed. Combining such studies produces a historical and setting-specific synthesis rather than an estimate of resistance at a single contemporary time point. This limitation is particularly important for ceftriaxone and cefixime because recent surveillance indicates rapid changes in cephalosporin resistance in some Asia-Pacific settings [8,27,28,29].

Substantial heterogeneity was observed for five of the six antibiotic outcomes. Because each analysis contained only three to five independent studies, the sources of heterogeneity could not be investigated reliably through subgroup analysis or meta-regression. The pooled estimates consequently average across important differences in country, surveillance period, clinical population, AST method, and breakpoint standard.

Sparse events and zero-event datasets affected the cefixime and ceftriaxone analyses. For cefixime, the model-based estimate differed markedly from the aggregate proportion and did not adequately summarize the observed distribution of resistance. The cefixime result should therefore be regarded as statistically unstable, with primary emphasis placed on individual study estimates and resistant/tested counts. Ceftriaxone resistance events were also concentrated in the recent EGASP dataset, resulting in substantial temporal and geographic heterogeneity.

Restricting eligibility to male urethral or urogenital isolates improved alignment with the clinical review question but excluded otherwise informative surveillance studies that did not report data separately by sex and anatomical site. Similarly, reports presenting MIC distributions without extractable categorical resistance counts could not contribute to the proportion meta-analysis. These restrictions improved comparability of the quantitative outcomes but reduced geographic coverage and may have excluded early evidence of shifts in susceptibility.

The small number of studies also prevented reliable assessment of publication bias and small-study effects. Moreover, surveillance studies can be affected by selection bias because isolates submitted for culture and AST may disproportionately represent symptomatic patients, referral populations, suspected treatment failures, or sites with stronger laboratory capacity. Finally, all six outcomes were rated as very low certainty, reflecting the combined effects of inconsistency, imprecision, sparse events, and limited study numbers. The pooled estimates should consequently be treated as exploratory and should not replace contemporary local surveillance.

## 5. Conclusions

Published evidence reporting antimicrobial resistance specifically in male urethral or urogenital *Neisseria gonorrhoeae* isolates remains limited and geographically uneven. Resistance to ciprofloxacin and penicillin was generally high but varied substantially across settings. The addition of contemporary EGASP evidence demonstrated that ceftriaxone resistance was not uniformly low and that older studies may not represent resistance patterns in current high-resistance settings.

Cefixime results were particularly sensitive to zero-event studies and the concentration of resistant isolates in a small number of large datasets. The antibiotic-specific pooled estimates should therefore be regarded as exploratory summaries rather than contemporary global prevalence values or an independent basis for empirical treatment selection.

The review identifies an important surveillance-reporting gap. Routine reporting of resistant and tested counts, MIC distributions, breakpoint standards and versions, sex, anatomical specimen site, clinical presentation, country, and surveillance period would substantially improve the interpretability and synthesis of gonococcal AMR evidence. Timely integration of locally relevant resistance data into treatment guidance remains essential for detecting emerging resistance and preserving the effectiveness of available therapies.

## Figures and Tables

**Table 1:** Characteristics of studies included in the systematic review and meta-analysis.

Author, Year	Country/WHO Region	AST Method	Gender	Number of *N. gonorrhoeae* Isolates	Antibiotics	Resistant
Zachariah, 2003 [20]	Malawi/AFRO	Disc diffusion method on agar	Hetero men	91	Gentamicin	7
				91	Penicillin	43
				91	Tetracycline	38
				91	Erythromycin	23
				91	Co-trimoxazole	26
				91	Spectinomycin	5
				91	Ciprofloxacin	3
Hançali, 2013 [21]	Morocco/EMRO	E-test method on agar dilution	Men	82	Penicillin	40
				82	Ceftriaxone	0
				82	Cefixime	0
				82	Ciprofloxacin	61
				82	Tetracycline	66
				82	Spectinomycin	0
Fentaw, 2020 [22]	Ethiopia/AFRO	Disc diffusion method on agar dilution	Men	415	Penicillin	191
				415	Spectinomycin	11
				415	Ciprofloxacin	217
				415	Ceftriaxone	0
				415	Cefixime	0
				415	Cefoxitin	4
				415	Azithromycin	36
Workneh, 2020 [23]	Uganda/AFRO	Disc diffusion method on agar dilution	Men	395	Cefixime	0
				397	Ceftriaxone	2
				399	Ciprofloxacin	399
				399	Penicillin G	364
				34	Spectinomycin	0
				399	Tetracycline	397
Jacobsson, 2021 [24]	EU/EEA	E-test method on agar dilution	Hetero men	3343	Ceftriaxone	5
				3307	Cefixime	196
				3302	Azithromycin	284
				3343	Ciprofloxacin	1921
			MSM	1928	Ceftriaxone	0
				1917	Cefixime	57
				1921	Azithromycin	134
				1926	Ciprofloxacin	900
			Males other/UNK	4621	Ceftriaxone	12
				4618	Cefixime	222
				4619	Azithromycin	334
				4613	Ciprofloxacin	265
EGASP, 2025 [8]	Multiple countries	E-test method on agar dilution	Men	2420	Azithromycin	98
				2420	Ceftriaxone	125
				2420	Cefixime	273

Abbreviations: MSM—Men who have sex with men; UNK—Unknown.

**Table 2:** Leave-one-out sensitivity analysis.

Analysis	Omitted	Pooled ES	Lower 95CI	Upper 95CI	I^2^ (%)
Azithromycin	Fentaw, 2020 [22]	−2.82	−3.29	−2.35	97.3
Azithromycin	Jacobsson, 2021 [24]	−2.79	−3.35	−2.23	93.8
Azithromycin	EGASP, 2025 [8]	−2.49	−2.56	−2.41	0
Cefixime	Hançali, 2013 [21]	−5.63	−9.84	−1.41	97.7
Cefixime	Fentaw, 2020 [22]	−5.00	−8.57	−1.44	97.7
Cefixime	Workneh, 2020 [23]	−5.03	−8.63	−1.44	97.7
Cefixime	Jacobsson, 2021 [24]	−8.46	−16.62	−0.3	0
Cefixime	EGASP, 2025 [8]	−8.24	−15.28	−1.20	0
Ceftriaxone	Hançali, 2013 [21]	−5.49	−7.41	−3.58	98
Ceftriaxone	Fentaw, 2020 [22]	−5.18	−6.89	−3.48	98
Ceftriaxone	Workneh, 2020 [23]	−6.06	−8.90	−3.21	98
Ceftriaxone	Jacobsson, 2021 [24]	−5.71	−8.54	−2.88	73
Ceftriaxone	EGASP,2025 [8]	−6.34	−6.79	−5.89	0
Ciprofloxacin	Zachariah, 2003 [20]	−2.00	−1.52	5.53	97
Ciprofloxacin	Hançali, 2013 [21]	−1.04	−3.60	5.70	97
Ciprofloxacin	Fentaw, 2020 [22]	−1.31	−3.34	5.95	96
Ciprofloxacin	Workneh, 2020 [23]	−0.72	−2.31	0.88	98
Ciprofloxacin	Jacobsson, 2021 [24]	1.52	−3.01	6.06	94
Penicillin	Zachariah, 2003 [20]	0.71	−0.60	2.02	99
Penicillin	Hançali, 2013 [21]	0.70	−0.63	2.02	99
Penicillin	Fentaw, 2020 [22]	0.74	−0.57	2.04	98
Penicillin	Workneh, 2020 [23]	−0.14	−0.30	0.02	0
Spectinomycin	Zachariah, 2003 [20]	−3.86	−4.45	−3.26	0
Spectinomycin	Hançali, 2013 [21]	−3.49	−3.99	−2.99	0
Spectinomycin	Fentaw, 2020 [22]	−4.99	−9.08	−0.91	0
Spectinomycin	Workneh, 2020 [23]	−3.58	−4.07	−3.08	0

**Table 3:** Evaluation of the certainty of evidence using GRADE for antibiotic resistance in *N. gonorrhoeae*.

Outcome	Study Design	Risk of Bias	Inconsistency	Indirectness	Imprecision	Publication Bias	Certainty of Evidence
Ceftriaxone resistance	Meta-analysis of observational and surveillance studies	Not serious	Serious	Not serious	Serious	Not assessed, fewer than 10 studies	Very low ⨁◯◯◯
Cefixime resistance	Meta-analysis of observational and surveillance studies	Not serious	Serious	Not serious	Very serious	Not assessed, fewer than 10 studies	Very low ⨁◯◯◯
Ciprofloxacin resistance	Meta-analysis of observational studies	Not serious	Serious	Not serious	Very serious	Not assessed, fewer than 10 studies	Very low ⨁◯◯◯
Penicillin resistance	Meta-analysis of observational studies	Not serious	Serious	Not serious	Not serious	Not assessed, fewer than 10 studies	Very low ⨁◯◯◯
Spectinomycin resistance	Meta-analysis of observational studies	Not serious	Not serious	Not serious	Serious	Not assessed, fewer than 10 studies	Very low ⨁◯◯◯
Azithromycin resistance	Meta-analysis of observational and surveillance studies	Not serious	Serious	Not serious	Not serious	Not assessed, fewer than 10 studies	Very low ⨁◯◯◯

Note: Filled circles/symbols (⨁) indicate retained certainty levels, while empty circles (◯) indicate downgraded levels.

## Data Availability

The original contributions presented in this study are included in the article. Further inquiries can be directed to the corresponding author.

## Supplementary Materials

The following supporting information can be downloaded at: https://www.mdpi.com/article/10.3390/medicina62081457/s1. Supplemental Table S1: Search strategy for the systematic review, Supplemental Table S2: Inclusion and exclusion criteria of study selection based on the PICOS framework; Supplemental Table S3: PRISMA Checklist; Supplemental Table S4: Quality of included studies using JBI critical appraisal tool.

## Abbreviations

The following abbreviations are used in this manuscript:

AFRO	WHO African Region
AMR	Antimicrobial resistance
AST	Antimicrobial susceptibility testing
CI	Confidence interval
EECA	Eastern Europe and Central Asia
EGASP	Enhanced Gonococcal Antimicrobial Surveillance Programme
EMRO	WHO Eastern Mediterranean Region
EU/EEA	European Union/European Economic Area
JBI	Joanna Briggs Institute
MIC	Minimum inhibitory concentration
MSM	Men who have sex with men
NOS	Newcastle-Ottawa Scale
PRISMA	Preferred Reporting Items for Systematic Reviews and Meta-Analyses
WHO	World Health Organization
UNK	Unknown

## References

[1] Unemo M., Lahra M.M., Escher M., Eremin S., Cole M.J., Galarza P., Ndowa F., Martin I., Dillon J.R., Galas M. (2021). WHO global antimicrobial resistance surveillance for Neisseria gonorrhoeae 2017-18: A retrospective observational study. Lancet Microbe.

[2] World Health Organization Gonorrhoea. WHO Fact Sheets. https://www.who.int/news-room/fact-sheets/detail/gonorrhoea.

[3] Unemo M., Seifert H.S., Hook E.W., Hawkes S., Ndowa F., Dillon J.-A.R. (2019). Gonorrhoea. Nat. Rev. Dis. Prim..

[4] Dallabetta G., Hook E.W. (1987). Gonococcal infections. Infect. Dis. Clin. N. Am..

[5] Parczewski M., Gökengin D. (2024). The HIV epidemic in eastern Europe and central Asia in difficult times: A story of resilience and change. J. Int. AIDS Soc..

[6] Unemo M., Shafer W.M. (2011). Antibiotic resistance in Neisseria gonorrhoeae: Origin, evolution, and lessons learned for the future. Ann. N. Y. Acad. Sci..

[7] World Health Organization (2016). WHO Guidelines for the Treatment of Neisseria Gonorrhoeae.

[8] Enhanced Gonococcal Antimicrobial Surveillance Programme (EGASP): Gonorrhoea Treatment Optimization, 2025 Report; ISBN: 978-92-4-011729-7. https://www.who.int/publications/i/item/9789240117297.

[9] Wi T., Lahra M.M., Ndowa F., Bala M., Dillon J.R., Ramon-Pardo P., Eremin S.R., Bolan G., Unemo M. (2017). Antimicrobial resistance in Neisseria gonorrhoeae: Global surveillance and a call for international collaborative action. PLoS Med..

[10] Centers for Disease Control and Prevention Antibiotic-Resistant Gonorrhea. https://www.cdc.gov/gonorrhea/hcp/drug-resistant/index.html.

[11] Hooshiar M.H., Sholeh M., Beig M., Azizian K., Kouhsari E. (2024). Global trends of antimicrobial resistance rates in Neisseria gonorrhoeae: A systematic review and meta-analysis. Front. Pharmacol..

[12] Fifer H., Saunders J., Soni S., Sadiq S.T., FitzGerald M. (2020). 2018 UK national guideline for the management of infection with Neisseria gonorrhoeae. Int. J. STD AIDS.

[13] Kirkcaldy R.D., Weston E., Segurado A.C., Hughes G. (2019). Epidemiology of gonorrhoea: A global perspective. Sex. Health.

[14] National Institute for Health and Care Research PROSPERO International Prospective Register of Systematic Reviews. https://www.crd.york.ac.uk/prospero/.

[15] Page M.J., McKenzie J.E., Bossuyt P.M., Boutron I., Hoffmann T.C., Mulrow C.D., Shamseer L., Tetzlaff J.M., Akl E.A., Brennan S.E. (2021). The PRISMA 2020 Statement: An Updated Guideline for Reporting Systematic Reviews. BMJ.

[16] Posit Team RStudio: Integrated Development Environment for R. http://www.posit.co/.

[17] Brown L.B., Krysiak R., Kamanga G., Mapanje C., Kanyamula H., Banda B., Mhango C., Hoffman M., Kamwendo D., Hobbs M. (2010). Neisseria gonorrhoeae antimicrobial susceptibility in Lilongwe, Malawi, 2007. Sex. Transm. Dis..

[18] Unemo M., Jensen J.S. (2017). Antimicrobial-resistant sexually transmitted infections: Gonorrhoea and Mycoplasma genitalium. Nat. Rev. Urol..

[19] Calado J., Castro R., Lopes Â., Campos M.J., Rocha M., Pereira F. (2019). Antimicrobial resistance and molecular characteristics of Neisseria gonorrhoeae isolates from men who have sex with men. Int. J. Infect. Dis..

[20] Zachariah R., Harries A.D., Nkhoma W., Arendt V., Nchingula D., Chantulo A., Chimtulo F., Kirpach P. (2003). Behavioural characteristics, prevalence of Chlamydia trachomatis and antibiotic susceptibility of Neisseria gonorrhoeae in men with urethral discharge in Thyolo, Malawi. Malawi Med. J..

[21] Hançali A., Ndowa F., Bellaji B., Bennani A., Kettani A., Charof R., El Aouad R. (2013). Antimicrobial resistance monitoring in Neisseria gonorrhoeae and strategic use of funds from the Global Fund to set up a systematic Moroccan gonococcal antimicrobial surveillance programme. Sex. Transm. Infect..

[22] Fentaw S., Abubeker R., Asamene N., Assefa M., Bekele Y., Tigabu E. (2020). Antimicrobial susceptibility profile of Gonococcal isolates obtained from men presenting with urethral discharge in Addis Ababa, Ethiopia: Implications for national syndromic treatment guideline. PLoS ONE.

[23] Workneh M., Hamill M.M., Kakooza F., Mande E., Wagner J., Mbabazi O., Mugasha R., Kajumbula H., Walwema R., Zenilman J. (2020). Antimicrobial Resistance of Neisseria Gonorrhoeae in a Newly Implemented Surveillance Program in Uganda: Surveillance Report. JMIR Public Health Surveill..

[24] Jacobsson S., Cole M.J., Spiteri G., Day M., Unemo M., Euro-GASP Network (2021). Associations between antimicrobial susceptibility/resistance of Neisseria gonorrhoeae isolates in European Union/European Economic Area and patients’ gender, sexual orientation and anatomical site of infection, 2009–2016. BMC Infect. Dis..

[25] Golparian D., Cole M.J., Sánchez-Busó L., Day M., Jacobsson S., Uthayakumaran T., Abad R., Bercot B., Caugant D.A., Heuer D. (2024). Antimicrobial-resistant Neisseria gonorrhoeae in Europe in 2020 compared with in 2013 and 2018: A retrospective genomic surveillance study. Lancet Microbe.

[26] Golparian D., Bazzo M.L., Ahlstrand J., Schörner M.A., Gaspar P.C., de Melo Machado H., Martins J.M., Bigolin A., Ramos M.C., Ferreira W.A. (2024). Recent dynamics in Neisseria gonorrhoeae genomic epidemiology in Brazil: Antimicrobial resistance and genomic lineages in 2017–20 compared with 2015–16. J. Antimicrob. Chemother..

[27] Xiu L., Zhang L., Peng J. (2024). Surge in ceftriaxone-resistant Neisseria gonorrhoeae FC428-like strains, Asia-Pacific region, 2015–2022. Emerg. Infect. Dis..

[28] Laumen J.G.E., Hieu V.N., Nhung P.H., Vandelannoote K., Nguyen T.T., Nguyen T.T., Nguyen D.T.N., Kesteman T., van Doorn H.R., Chau T.M. (2025). High prevalence of ceftriaxone-resistant Neisseria gonorrhoeae in Hanoi, Vietnam, 2023–2024. J. Infect. Dis..

[29] Fifer H., Doumith M., Rubinstein L., Mitchell L., Wallis M., Singh S., Singh G.J., Rayment M., Evans-Jones J., Blume A. (2024). Ceftriaxone-resistant Neisseria gonorrhoeae detected in England, 2015–2024: An observational analysis. J. Antimicrob. Chemother..

[30] Quilter L.A.S., Cyr S.B.S., Barbee L.A. (2024). The management of gonorrhea in the era of emerging antimicrobial resistance: What primary care clinicians should know. Med. Clin. N. Am..

[31] Allan-Blitz L.T., Fifer H., Klausner J.D. (2024). Managing treatment failure in Neisseria gonorrhoeae infection: Current guidelines and future directions. Lancet Infect. Dis..

[32] Unemo M., Sánchez-Busó L., Golparian D., Jacobsson S., Shimuta K., Lan P.T., Eyre D.W., Cole M., Maatouk I., Wi T. (2024). The novel 2024 WHO Neisseria gonorrhoeae reference strains for global quality assurance of laboratory investigations and superseded WHO *N. gonorrhoeae* reference strains: Phenotypic, genetic and reference genome characterization. J. Antimicrob. Chemother..

