# Supplementary material for: Antimicrobial Resistance in Neisseria gonorrhoeae Isolates from Men with Urethral or Urogenital Infection: A Systematic Review and Meta-Analysis
Source: Medicina (Kaunas). 2026 Jul 28;62(8):1457. doi: 10.3390/medicina62081457 (PMC13514914; doi:10.3390/medicina62081457)
Supplement: Supplementary file 1 [file medicina-62-01457-s001.zip › Supplemental Table S1.pdf]

Supplemental Table S1. Search strategy for the systematic review.

Database	Search Fields	Filters
PubMed	Title, Abstract, Keywords	Language: English<br><br>Species: Human
Science Direct	Title, Abstract, Keywords	Language: English
Scopus	Title, Abstract, Keywords	Language: English<br><br>Document Type: Articles
Google Scholar	Title	Language: English
WHO EGASP publications	Title	Language: English
