# Supplementary material for: Antimicrobial Resistance in Neisseria gonorrhoeae Isolates from Men with Urethral or Urogenital Infection: A Systematic Review and Meta-Analysis
Source: Medicina (Kaunas). 2026 Jul 28;62(8):1457. doi: 10.3390/medicina62081457 (PMC13514914; doi:10.3390/medicina62081457)
Supplement: Supplementary file 1 [file medicina-62-01457-s001.zip › Supplemental Table S2.pdf]

Supplemental Table S2. Inclusion and exclusion criteria of study selection based on the PICOS framework.

**PICOS framework**	**Inclusion criteria**	**Exclusion criteria**
Population	Studies that included male participants from whom Neisseria gonorrhoeae was isolated using urogenital specimens (e.g., urethral swabs or urine samples)	Studies that did not report male-specific data; included only female or mixed populations without stratification; used only non-urogenital specimens
Intervention	Not applicable	Not applicable
Comparator	Not applicable	Not applicable
Outcome	Studies reporting antimicrobial susceptibility and/or resistance outcomes, including resistance rates (%), MIC values, or susceptibility categories (S/I/R), with extractable data for at least one antibiotic	Studies without AMR/AST outcomes; lacking extractable data (e.g., missing denominators); reporting only molecular data without phenotypic susceptibility; focusing only on clinical outcomes
Study design	Cross-sectional, prospective or retrospective observational studies, surveillance reports, cohort and case–control studies reporting AMR data	Reviews, editorials, commentaries, case reports/series, conference abstracts without full data, studies with unclear or non-standard AST methods and studies published in languages other than English
