# Supplementary material for: Antimicrobial Resistance in Neisseria gonorrhoeae Isolates from Men with Urethral or Urogenital Infection: A Systematic Review and Meta-Analysis
Source: Medicina (Kaunas). 2026 Jul 28;62(8):1457. doi: 10.3390/medicina62081457 (PMC13514914; doi:10.3390/medicina62081457)
Supplement: Supplementary file 1 [file medicina-62-01457-s001.zip › Supplemental Table S3.pdf]

PRISMA Checklist

Section and Topic	Item #	Checklist item	Location where item is reported
**TITLE**			
Title	1	Identify the report as a systematic review.	Page 1, title, lines 1-4.
**ABSTRACT**			
Abstract	2	See the PRISMA 2020 for Abstracts checklist.	Page 1, Abstract, lines 19-44; Page 2, Abstract continuation, lines 45-50.
**INTRODUCTION**			
Rationale	3	Describe the rationale for the review in the context of existing knowledge.	Page 2, Introduction, lines 53-93; Page 3, Introduction continuation, lines 94-100.
Objectives	4	Provide an explicit statement of the objective(s) or question(s) the review addresses.	Page 3, final Introduction paragraph/objectives, lines 101-108.
**METHODS**			
Eligibility criteria	5	Specify the inclusion and exclusion criteria for the review and how studies were grouped for the syntheses.	Page 3, Section 2.2 Eligibility Criteria, lines 121-144; Page 4, continuation and Section 2.4 Outcome Measures, lines 145-199.
Information sources	6	Specify all databases, registers, websites, organisations, reference lists and other sources searched or consulted to identify studies. Specify the date when each source was last searched or consulted.	Page 3, Section 2.1 Search Strategy, lines 110-120.
Search strategy	7	Present the full search strategies for all databases, registers and websites, including any filters and limits used.	Page 3, Section 2.1 Search Strategy, lines 110-120; Supplementary Table S1.
Selection process	8	Specify the methods used to decide whether a study met the inclusion criteria of the review, including how many reviewers screened each record and each report retrieved, whether they worked independently, and if applicable, details of automation tools used in the process.	Page 4, Section 2.3 Study Selection and Data Collection, lines 145-178.
Data collection process	9	Specify the methods used to collect data from reports, including how many reviewers collected data from each report, whether they worked independently, any processes for obtaining or confirming data from study investigators, and if applicable, details of automation tools used in the process.	Page 4, Section 2.3 Study Selection and Data Collection, lines 154-178.
Data items	10a	List and define all outcomes for which data were sought. Specify whether all results that were compatible with each outcome domain in each study were sought (e.g. for all measures, time points, analyses), and if not, the methods used to decide which results to collect.	Page 4-5, Section 2.4 Outcome Measures, lines 179-199.
	10b	List and define all other variables for which data were sought (e.g. participant and intervention characteristics, funding sources). Describe any assumptions made about any missing or unclear information.	Page 4, Section 2.3 data extraction variables, lines 154-160; overlap/selection assumptions, lines 161-178.
Study risk of bias assessment	11	Specify the methods used to assess risk of bias in the included studies, including details of the tool(s) used, how many reviewers assessed each study and whether they worked independently, and if applicable, details of automation tools used in the process.	Page 5, Section 2.6 Quality Assessment and Certainty of Evidence, lines 222-245; Supplementary Table S4.
Effect measures	12	Specify for each outcome the effect measure(s) (e.g. risk ratio, mean difference) used in the synthesis or presentation of results.	Page 4-5, Section 2.4 Outcome Measures, lines 179-199; Page 5, Section 2.5 Statistical Analysis, lines 200-221.
Synthesis methods	13a	Describe the processes used to decide which studies were eligible for each synthesis (e.g. tabulating the study intervention characteristics and comparing against the planned groups for each synthesis (item #5)).	Page 3-5, Sections 2.2-2.4, lines 121-199.
	13b	Describe any methods required to prepare the data for presentation or synthesis, such as handling of missing summary statistics, or data conversions.	Page 4, Section 2.3 Data Collection, lines 154-178; Page 5, Section 2.5 Statistical Analysis, lines 200-215.

PRISMA Checklist

Section and Topic	Item #	Checklist item	Location where item is reported
	13c	Describe any methods used to tabulate or visually display results of individual studies and syntheses.	Page 8-9, Table 1; Page 10-12, Figure 2; Page 9-10, Section 3.2, lines 299-370.
	13d	Describe any methods used to synthesize results and provide a rationale for the choice(s). If meta-analysis was performed, describe the model(s), method(s) to identify the presence and extent of statistical heterogeneity, and software package(s) used.	Page 5, Section 2.5 Statistical Analysis, lines 200-221.
	13e	Describe any methods used to explore possible causes of heterogeneity among study results (e.g. subgroup analysis, meta-regression).	Page 5, Section 2.5 Statistical Analysis, lines 216-221; Page 17, Section 4.2 Limitations, lines 567-573.
	13f	Describe any sensitivity analyses conducted to assess robustness of the synthesized results.	Page 12-13, Table 2 and leave-one-out sensitivity analysis text, lines 372-397.
Reporting bias assessment	14	Describe any methods used to assess risk of bias due to missing results in a synthesis (arising from reporting biases).	Page 5, Section 2.5 Statistical Analysis, lines 217-221; Page 13, GRADE/reporting-bias note, lines 429-432.
Certainty assessment	15	Describe any methods used to assess certainty (or confidence) in the body of evidence for an outcome.	Page 5, Section 2.6 Quality Assessment and Certainty of Evidence, lines 234-245.
**RESULTS**			
Study selection	16a	Describe the results of the search and selection process, from the number of records identified in the search to the number of studies included in the review, ideally using a flow diagram.	Page 6, Section 3.1 Study Selection, lines 247-272; Figure 1 on Page 7.
	16b	Cite studies that might appear to meet the inclusion criteria, but which were excluded, and explain why they were excluded.	Page 6, Section 3.1 Study Selection, lines 257-266; excluded reports cited as applicable in References [17-19] and EGASP exclusion category.
Study characteristics	17	Cite each included study and present its characteristics.	Page 8-9, Table 1; included studies cited in References [8, 20-24].
Risk of bias in studies	18	Present assessments of risk of bias for each included study.	Page 5, Section 2.6, lines 222-245; Supplementary Table S4.
Results of individual studies	19	For all outcomes, present, for each study: (a) summary statistics for each group (where appropriate) and (b) an effect estimate and its precision (e.g. confidence/credible interval), ideally using structured tables or plots.	Page 8-9, Table 1; Page 9-12, Section 3.2 and Figure 2, lines 299-370.
Results of syntheses	20a	For each synthesis, briefly summarise the characteristics and risk of bias among contributing studies.	Page 8-9, Table 1; Page 13-14, certainty-of-evidence text and Table 3, lines 398-435.
	20b	Present results of all statistical syntheses conducted. If meta-analysis was done, present for each the summary estimate and its precision (e.g. confidence/credible interval) and measures of statistical heterogeneity. If comparing groups, describe the direction of the effect.	Page 9-12, Section 3.2 Meta-Analysis of Antibiotic Resistance and Figure 2, lines 299-370.
	20c	Present results of all investigations of possible causes of heterogeneity among study results.	Page 9-13, Section 3.2 reports heterogeneity for each antibiotic and leave-one-out patterns, lines 299-397; Page 17, Limitations, lines 567-573.

PRISMA Checklist

Section and Topic	Item #	Checklist item	Location where item is reported
	20d	Present results of all sensitivity analyses conducted to assess the robustness of the synthesized results.	Page 12-13, Table 2 and leave-one-out sensitivity analysis text, lines 372-397.
Reporting biases	21	Present assessments of risk of bias due to missing results (arising from reporting biases) for each synthesis assessed.	Formal reporting-bias assessment was not performed because fewer than ten studies contributed to each outcome; Page 5, Section 2.5, lines 217-221; Page 13, lines 429-432.
Certainty of evidence	22	Present assessments of certainty (or confidence) in the body of evidence for each outcome assessed.	Page 13-14, GRADE/certainty-of-evidence text and Table 3, lines 398-435.
**DISCUSSION**			
Discussion	23a	Provide a general interpretation of the results in the context of other evidence.	Page 14-16, Section 4.1 Principal Findings and Interpretation, lines 444-548.
	23b	Discuss any limitations of the evidence included in the review.	Page 17, Section 4.2 Limitations, lines 549-596.
	23c	Discuss any limitations of the review processes used.	Page 17, Section 4.2 Limitations, especially lines 573-596.
	23d	Discuss implications of the results for practice, policy, and future research.	Page 16, Discussion implications, lines 527-548; Page 18, Conclusions, lines 600-614.
**OTHER INFORMATION**			
Registration and protocol	24a	Provide registration information for the review, including register name and registration number, or state that the review was not registered.	Page 3, Section 2.1 Search Strategy, lines 110-112; PROSPERO registration CRD420261284374.
	24b	Indicate where the review protocol can be accessed, or state that a protocol was not prepared.	Page 3, Section 2.1 Search Strategy, lines 110-112; PROSPERO registration CRD420261284374 and Reference [14].
	24c	Describe and explain any amendments to information provided at registration or in the protocol.	Page 3, Section 2.1 Search Strategy, lines 110-120; updated search and targeted WHO EGASP supplementary search described.
Support	25	Describe sources of financial or non-financial support for the review, and the role of the funders or sponsors in the review.	Page 18, Funding statement, lines 614-615.
Competing interests	26	Declare any competing interests of review authors.	Page 18, Conflicts of Interest statement, line 623.
Availability of data, code and other materials	27	Report which of the following are publicly available and where they can be found: template data collection forms; data extracted from included studies; data used for all analyses; analytic code; any other materials used in the review.	Page 18, Data Availability Statement, lines 619-621.

From: Page MJ, McKenzie JE, Bossuyt PM, Boutron I, Hoffmann TC, Mulrow CD, et al. The PRISMA 2020 statement: an updated guideline for reporting systematic reviews. BMJ 2021;372:n71. doi: 10.1136/bmj.n71. This work is licensed under CC BY 4.0. To view a copy of this license, visit https://creativecommons.org/licenses/by/4.0/
