# Supplementary material for: Antimicrobial Resistance in Neisseria gonorrhoeae Isolates from Men with Urethral or Urogenital Infection: A Systematic Review and Meta-Analysis
Source: Medicina (Kaunas). 2026 Jul 28;62(8):1457. doi: 10.3390/medicina62081457 (PMC13514914; doi:10.3390/medicina62081457)
Supplement: Supplementary file 1 [file medicina-62-01457-s001.zip › Supplemental Table S4.pdf]

Supplemental Table S4: Quality of included studies using JBI critical appraisal tool

Authors	1	2	3	4	5	6	7	8	Score (100%)
	Were the criteria for inclusion in the sample clearly defined?	Were the study subjects and the setting described in detail?	Was the exposure measured in a valid and reliable way?	Were objective, standard criteria used for measurement of the condition?	Were confounding factors identified?	Were strategies to deal with confounding factors stated?	Were the outcomes measured in a valid and reliable way?	Was appropriate statistical analysis used?	
Zachariah et al., 2003	Yes	Yes	Yes	Yes	Yes	Yes	Yes	Yes	100
Hançali et al., 2013	Yes	Yes	Yes	Yes	Yes	Yes	Yes	Yes	100
Fentaw et al., 2020	Yes	Yes	Yes	Yes	Yes	Yes	Yes	Yes	100
Workneh et al., 2020	Yes	Yes	Yes	Yes	Yes	Yes	Yes	Yes	100
Jacobsson et al., 2021	Yes	Yes	Yes	Yes	Yes	Yes	Yes	Yes	100
EGASP, 2025	Yes	Yes	Yes	Yes	Yes	Yes	Yes	Yes	100
